# Tannins extract from *Galla Chinensis* can protect mice from infection by *Enterotoxigenic Escherichia coli* O101

**DOI:** 10.1186/s12906-021-03261-x

**Published:** 2021-03-06

**Authors:** Xu Song, Yi Yang, Junzhi Li, Mengxue He, Yuanfeng Zou, Renyong Jia, Lixia Li, Juan Hang, Min Cui, Lu Bai, Zhongqiong Yin

**Affiliations:** 1 Natural Medicine Research Center, College of Veterinary Medicine, Sichuan Agricultural University, Chengdu, 611130 China; 2 College of Veterinary Medicine, Sichuan Agricultural University, Chengdu, 611130 P. R. China

**Keywords:** *Galla Chinensis*, Enterotoxigenic *Escherichia coli*, Diarrhea, Tannins

## Abstract

**Background:**

Enterotoxigenic *Escherichia coli* (ETEC) is classically associated with acute secretory diarrhea, which induces 2 million people death in developing countries over a year, predominantly children in the first years of life. Previously, tannins (47.75%) were extracted from *Galla Chinensis* and prepared as *Galla Chinensis* oral solution (GOS) which showed significant antidiarrheal activity in a castor oil-induced diarrhea in mice. Whether the tannins extract were also effective in treatment of ETEC-induced diarrhea was determined in this study.

**Methods:**

Mice were randomly divided into 6 groups (*n* = 22). The mice in the normal and untreated groups were given normal saline. Three GOS-treated groups were received different concentrations of GOS (5, 10 and 15%, respectively) at a dose of 10 mL/kg. Mice in the positive control group were fed with loperamide (10 mg/kg). The treatment with GOS started 3 days before infection with ETEC and continued for 4 consecutive days after infection. On day 3, mice were all infected with one dose of LD_50_ of ETEC, except those in the normal group. Survival of mice was observed daily and recorded throughout the study. On days 4 and 7, samples were collected from 6 mice in each group.

**Results:**

GOS could increase the survival rate up to 75%, while in the untreated group it is 43.75%. The body weights of mice treated with 15% GOS were significantly increased on day 7 in comparison with the untreated group and the normal group. GOS-treatment recovered the small intestine coefficient enhanced by ETEC-infection. The diarrhea index of mice treated with GOS was significantly decreased. GOS increased the levels of IgG and sIgA in the terminal ileum and decreased the levels of pro-inflammatory cytokines (IFN-γ, TNF-α, IL-1β, IL-6 and IL-8) in serum. GOS could increase the amount of intestinal probiotics, *Lactobacilli* and *Bifidobacteria*. GOS could alleviate colon lesions induced by ETEC-infection. GOS showed higher potency than loperamide.

**Conclusions:**

GOS could be a promising drug candidate for treating ETEC infections.

## Background

Enterotoxigenic *Escherichia coli* (ETEC) are a diverse group of pathogens that have in common the ability to colonize the small intestine, which are classically associated with acute secretory diarrhea [1]. ETEC is a food and water-borne pathogen, typically ingested by contaminated food and drinking water; animals and humans are easily infected [2, 3]. Two major types of toxins, heat-labile enterotoxin and heat-stable enterotoxin, are secreted by ETEC, which activate the cystic fibrosis transmembrane conductance regulator (CFTR), and then leads to a net flow of water from the cell into the intestinal lumen, resulting in profuse watery diarrhea [4]. On any given day, an estimated 200 million people worldwide will suffer from gastroenteritis. Approximately 2 million of these people living in developing countries would die as a result of these diseases in a year, predominantly children in the first years of life [5]. Multiple pathogens are responsible for this suffering and death in developing countries, but ETEC is regarded as the most common bacterial cause of diarrhea [6]. There are six major serotypes of ETEC, including O6, O27, O101, O148, O149 and O159, and O101 is one of the commonly causative agents for diarrhea [7]. Thus, in the present study, the O101 strain is used to establish a mouse model of ETEC -induced diarrhea.

Antibacterial activity against *E. coli* is always related to antidiarrheal activity. Many medicinal plants were validated as treatments for ETEC-infected diarrhea on the basis of their ability to prevent or ameliorate diarrheal symptoms, such as anti-secretory and anti-motility activities [8, 9]. Magnolol and honokiol could inhibit ETEC O78-induced diarrhea by regulating the calcium-activated potassium channels [10]. *Chaenomeles speciosa* could inhibit *E. coli* heat-labile enterotoxin-induced diarrhea [11]. Most of these plants contain tannins. It was reported that tannins were one of the active compounds to resist *E. coil* [9]. An herbal medicine named modified *Pulsatilla* powder showed antibacterial activity against ETEC O101 infection in mice [12].

*Galla chinensis* contains a large amount of tannins [13]. It is the term used to describe the gall caused by the Chinese aphid (family *Pemphigidae*) on the *Rhus* leaves of the family *Anacardiaceae* (mainly *Rhus chinensis* Mill, *Rhus potaninii* Maxim, and *Rhus punjabensis var. sinica* (Diels) Rehd. et Wils) [14]. The medical uses of *Galla chinensis* are various, including diarrhea, inflammations, antibacterial and intestinal cancer [15]. It is reported that *Galla chinensis* extracts can inhibit various bacterial growth in vitro, such as periodontopathic bacteria [16], *Staphycoccus aureus* [17], *E. coli* [18] and intestinal bacteria [19]. Other studies showed *Galla chinensis* extract was effective on inactivation of ETEC enterotoxin [20].

In our previous study, tannins extracted from *Galla Chinensis* were prepared as oral solution (GOS), which contained the tannins extract, purified water, appropriate sweeteners and antioxidant. We found that tannins extract showed significant antidiarrheal activity in a castor oil-induced diarrhea model in mice [21]. The acute toxicity study showed that the GOS is safe under the dose of 2500 mg/kg, and the LD_50_ is 3247.47 mg/kg (Unpublished data). In the present study, whether tannins from *Galla Chinensis* also possessed antibacterial activity in the ETEC-infected mice model was tested for the purpose of developing a new antidiarrheal drug.

## Materials and methods

### Tannins extraction and preparation of *Galla chinensis* oral solution

The extraction process of tannins and preparation of *Galla chinensis* oral solution (GOS) was described in our previous report [21]. Briefly, tannins from *Galla chinensis* were extracted by boiling water, and purified by removal of impurities through chloroform extraction and ethylacetate extraction, respectively. The content of tannins was 47.75%. For convenient application, the tannin was prepared as GOS with the tannins extract, purified water, appropriate excipients (sucrose, benzoic acid, ethyl p-hydroxybenzoate and natrium pyrosulfurosum). The GOS containing different concentrations of tannins extract (5, 10 and 15%) were used in this study.

### Animals

Four-week-old male ICR mice (Average body weight of 20 ± 2 g) were purchased from Chengdu Dossy Experimental Animals Co., Ltd. [License No. SCXK (Sichuan) 2015–030]. The mice were reared in polypropylene cages, for each cage with no more than 5 mice. Mice were fed normally with NIH-07 standard diets (TROPHIC Animal Feed High-tech Co Ltd., China) in animal house at controlled temperature (23 ± 2 °C), with alternating 12 h periods of light and dark. It took 7 days to make animals get acclimatized to the new conditions.

Animals were monitored at least once daily prior to infection and at least twice daily after infection. Any animal, body weight loss above 15% of initial weight [22], showing signs of severe and enduring distress or characterized as moribund [23], was considered as dead, and then was euthanized by cervical dislocation. 34 of total 150 mice (22.6%) in these studies did succumb prior to administration of euthanasia; this was within the 30% anticipated in the approved protocols.

### Bacteria strains

Standard *E. coli* strains (CVCC3749, serotype O101) were bought from China Veterinary Culture Collection Center (Beijing, China). The bacteria was grown at 37 °C in Mueller-Hinton Broth (MHB) cultures overnight and suspensions were determined with a turbidity equivalent to that of a 3 McFarland standard (10^8^ CFU / mL).

### Determination of *E. coli* O101 LD_50_ in mice

LD_50_ of *E. coli* O101 was calculated using a dose–response method [24]. Twenty-four male mice were randomly divided into six groups. All of them fasted for 6 h before treatment. Animals in different groups were challenged through intraperitoneal injection with approximately 1.78 × 10^8^, 1.11 × 10^9^, 2.18 × 10^9^, 4.47 × 10^9^, 8.91 × 10^9^, 1.77 × 10^10^ CFU of *E. coli* O101 at a volume of 0.2 mL per mouse, respectively [25, 26]. Control group received equal volume of normal saline. After infection, the animals were observed for behavioral changes, signs of toxicity and death during the first 12 h and thereafter twice a day for 14 days. Mice that stay alive throughout 14 days’ observation were recorded as survivors. The number of dead mice at each dose were used to calculate the LD_50_ according to Karber’s method [27] and a dosage-mortality curve were obtained using the GraphPad Prism software (SanDiego, CA, USA).

### Antibacterial test in mice

#### Experimental design

Animals were randomly divided into 6 groups (*n* = 22). Before pre-treatment, mice were fasted for 6 h. The mice in the normal and untreated controls were given normal saline. The three GOS groups were received different concentrations of GOS (5, 10 and 15%, respectively) at a dose of 10 mL/kg. Mice in the positive control group were fed with loperamide (10 mg/kg) which suspended in distilled water [28]. Treatment with GOS started 3 days before infection and continued for 4 consecutive days after infection. On day 3, after weighed, mice were all infected with one dose of LD_50_ of ETEC, except those in the normal group. Survival of mice was observed daily throughout the study. On day 4, 6 mice in each group were used for blood samples collection through retro orbital puncture under anaesthesia by isoflurane inhalation. In order to minimize animals suffering during blood samples collection, an eye drop of tetracaine 1% was applied [22]. Then, the animals were subjected to a full necropsy. The cecum contents were collected into sterile plastic tubes and stored at − 80 °C until analysis. On day 7, 6 mice were randomly chosen from the survival mice for sample collection as described above.

#### Diarrhea index

After infection, the numbers and morphology of the stools were observed daily. The loose stool incidence rate (LSIR) is calculated as the ratio of number of loose stools to the total stools within an animal. The loose stool grade (LSG) is the degree of loose stools which was classified into four grades according the diameter of loose stools on the filter papers: Grade 1 (< 1 cm), Grade 2 (1–2 cm), Grade 3 (2–3 cm), and Grade 4 (> 3 cm). The average loose stool grade (ALSG) is calculated as the ratio of the sum of LSG of each loose stool to the total number of loose stools within an animal. The diarrhea index is calculated by multiplying LSIR with ALSG [12].

#### Body weight

Body weights of all mice were constantly monitored every day throughout the study.

#### Survival rate

Animals were observed daily throughout the experiment and percentage of survival mice was recorded. The survival rate in each group was calculated using the following formula [25]:

$$ \mathrm{survival}\ \mathrm{rate}=\frac{\mathrm{Number}\ \mathrm{of}\ \mathrm{surviving}\ \mathrm{mice}\ \mathrm{after}\ \mathrm{challenge}}{\mathrm{Number}\ \mathrm{of}\ \mathrm{mice}\ \mathrm{injected}\ \mathrm{with}\ \mathrm{bacteria}}\times 100\% $$

#### Organ coefficient assay

Liver, spleen, and small intestine of animals were weighted. The relative weight of organs (Organ coefficient) was calculated using the following formula [29]:

$$ \mathrm{Organ}\ \mathrm{coefficients}=\mathrm{Organ}\ \mathrm{weight}\ \left(\mathrm{g}\right)/\mathrm{Body}\ \mathrm{weight}\ \left(\mathrm{g}\right)\times 100\% $$

#### Levels of cytokines and immunoglobulins

The concentrations of 6 cytokines (IFN-γ, TNF-α, IL-1β, IL-4, IL-6 and IL-8 in serum) and IgG in serum and sIgA in the terminal ileum were measured by enzyme-linked immunosorbent assay (ELISA) with commercially available ELISA kits (MLBIO Biolotechnology Co. Ltd., Shanghai, China). ELISA was performed according to the manufacturer’s instructions. All the samples were assayed in duplicate and the absorbance values were analyzed by non-linear regression.

#### Determination of selected cecum microbiota

The dilution plate counting method was used to evaluate the regulatory effect of GOS on the intestinal flora in mice. The fresh stool samples in the cecum were collected, serially diluted, and plated on MRS, TPY, VRBA, and Kanamycin Aesculin Azide agar (Qingdao Hope Bio-Technology Co. Ltd., China) to identify and count the number of *Lactobacilli*, *Bifidobacteria*, *E. coli*, *Enterococcus*, respectively [12].

#### Histopathological examination

The colon was preserved in 4% paraformaldehyde. After gradient dehydration and enclosed in paraffin, samples were cut into 5 μm thick serial sections for hematoxylin and eosin (HE) staining. The lesions were observed under optical microscope [26].

### Statistical analysis

The statistical significance was compared by one way analysis of variance (ANOVA) followed by the Student-Newman-Keuls test using the IBM SPSS Statistics, version 24 (NY, USA). The differences between groups would be considered significant when *p*-value is less than 0.05.

## Results

### Determination of *E. coli* O101 LD_50_

**Fig. 1:**
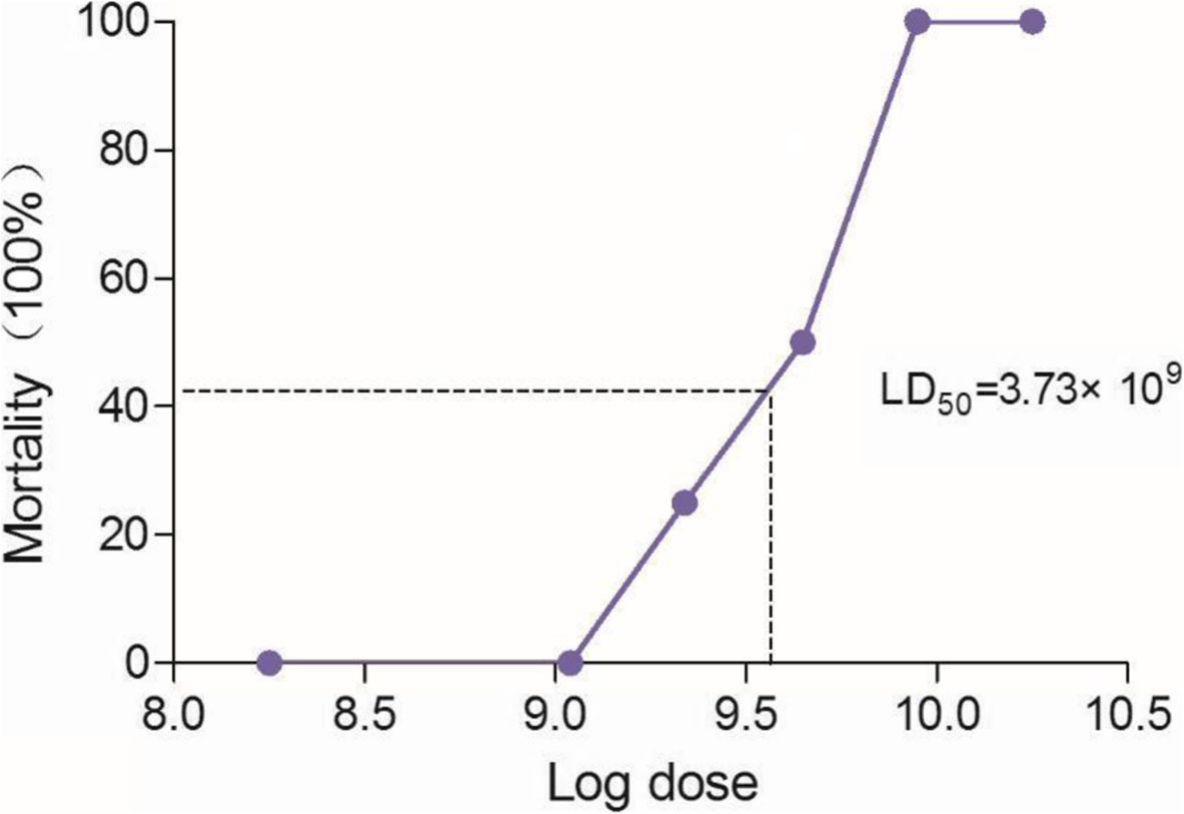
The dosage-mortality curve of *E. coli* O 101 in ICR mice

As shown in Fig. 1, it was found that the *E. coli* O101 at the dose of 2.18 × 10^9^ CFU caused death, and at the dose of over 8.89 × 10^9^ CFU induced 100% mortality. The LD_50_ of the *E. coli* O101 was determined to be 3.73 × 10^9^ CFU in male ICR mice. Therefore, for all subsequent experiments, mice were infected with LD_50_ dose of 3.73 × 10^9^ CFU.

### Survival rate

The results were listed in Table 1. The mice infected with ETEC survived less than half (43.75%). The survival rate in the loperamide group is 62.50%. Treated with 10 and 15% GOS, the survival rates were 68.75 and 75%, respectively, which are higher than loperamide. GOS could dose-dependently enhance the survival rate of ETEC-infected mice.

**Table 1:** Survival of mice after challenge with the LD_50_ of *E. coli* O101 on day 7

Group	Total number ^a^	Survivor number	Survival rate(%)
Normal	16	16	100
Negative control	16	7	43.75
Loperamide	16	10	62.50
GOS 5%	16	9	56.25
GOS 10%	16	11	68.75
GOS 15%	16	12	75.00

^a^ Total number of mice was excluded the 6 killed mice on day 4. Normal, the uninfected-untreated control. Negative control, the infected-untreated control. Loperamide, the infected group with Loperamide-treatment (10 mg/kg). The 5, 10 and 15% GOS were the infected groups treated with corresponding concentration of GOS at a dose of 10 mL/kg, respectively

### Diarrhea index

ETEC infection induced clinical diarrhea in mice (Table 2). On day 4, all the infected groups showed diarrhea, and the diarrhea index of GOS (10 and 15%) and loperamide groups were significantly lower than that of the untreated group (*P* < 0.05). The diarrhea index of the 15% GOS group is significantly lower than that of other GOS groups (*P* < 0.05). On day 5, the diarrhea index of all the treated-groups were significantly decreased when compared with the untreated group (*P* < 0.05). On day 6, the mice in the GOS and loperamide groups were recovered from diarrhea. The ETEC-infected mice without treatment did not show diarrhea until day 7. The anti-diarrhea activity of GOS showed a dose-dependent manner.

**Table 2:** The diarrhea index in each group

Group	Day 4	Day 5	Day 6	Day 7
Normal	0.00 ± 0.00	0.00 ± 0.00	0.00 ± 0.00	0.00 ± 0.00
Negative control	2.87 ± 0.59	1.26 ± 0.31	0.25 ± 0.16	0.00 ± 0.00
Loperamide	1.51 ± 0.52 ^*^	0.11 ± 0.09 ^**^	0.00 ± 0.00^*^	0.00 ± 0.00
GOS 5%	2.06 ± 0.24 ^a^	0.22 ± 0.24 ^**^	0.00 ± 0.00^*^	0.00 ± 0.00
GOS 10%	1.64 ± 0.41 ^*a^	0.15 ± 0.04 ^**^	0.00 ± 0.00^*^	0.00 ± 0.00
GOS 15%	1.18 ± 0.33 ^**b^	0.06 ± 0.04 ^**^	0.00 ± 0.00^*^	0.00 ± 0.00

Normal, the uninfected-untreated control. Negative control, the infected-untreated control. Loperamide, the infected group with Loperamide-treatment (10 mg/kg). The 5, 10 and 15% GOS were the infected groups treated with corresponding concentration of GOS at a dose of 10 mL/kg, respectively. ^*^ *P* < 0.05 and ^**^ *P* < 0.01, compared with the negative control. ^a, b^ Different letters indicate significant differences existed among the three GOS groups (*P* < 0.05). Data are presented as mean ± S.E.M. *n* = 16

### Body weight

**Fig. 2:**
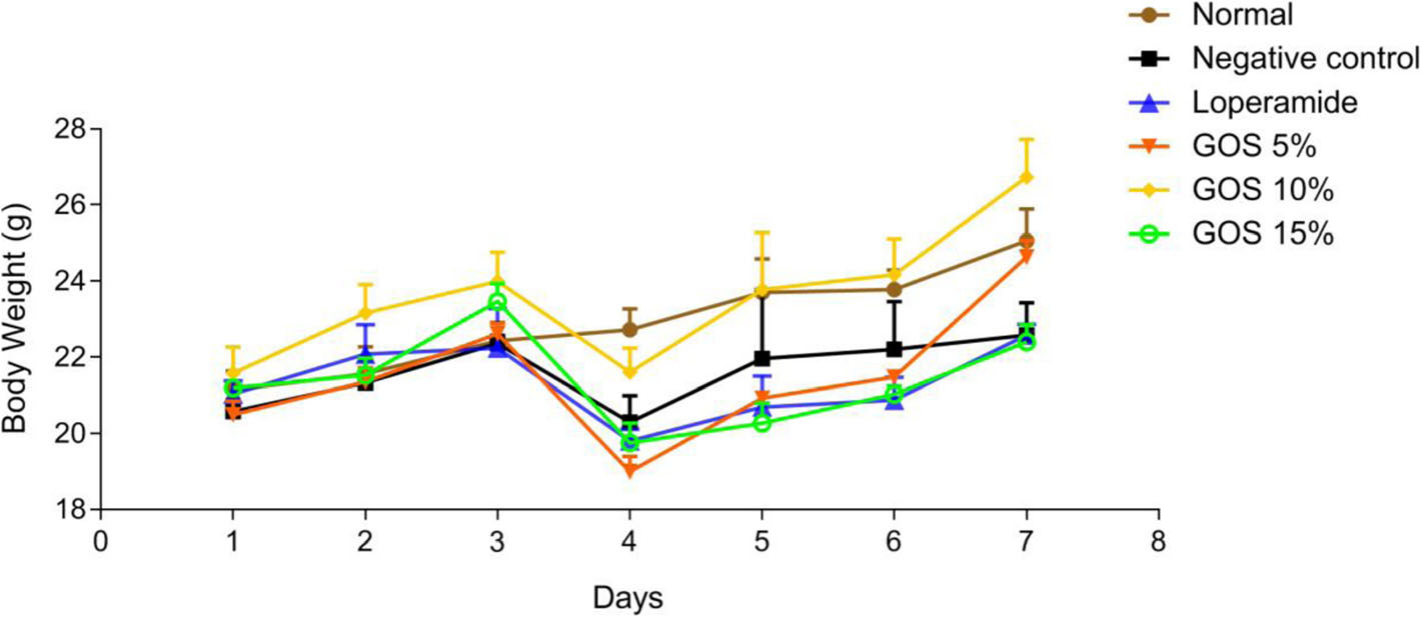
The body weight of mice from day 1 to day 7. Data are presented as mean ± S.E.M, *n* = 6. Normal, the uninfected-untreated control. Negative control, the infected-untreated control. Loperamide, the infected group with Loperamide-treatment (10 mg/kg). The 5, 10 and 15% GOS were the infected groups treated with corresponding concentration of GOS at a dose of 10 mL/kg, respectively

The body weights of mice in each group during the test were displayed in Fig. 2. The body weight showed an upward trend and no significant difference was detected in all mice before infection. Without treatment, the body weight of mice was significantly declined after infection on day 4 (*P* < 0.05). Compared with the untreated group, 10% GOS could significantly increase the weight of mice on day 4 (*P* < 0.05); the body weight is almost the same as the normal mice on day 5. The weight gain of mice in the 10% GOS-treated group was significantly (*P* < 0.05) higher than that in other treated groups (loperamide, 5 and 15% GOS) after infection.

### Relative weight of organs

**Fig. 3:**
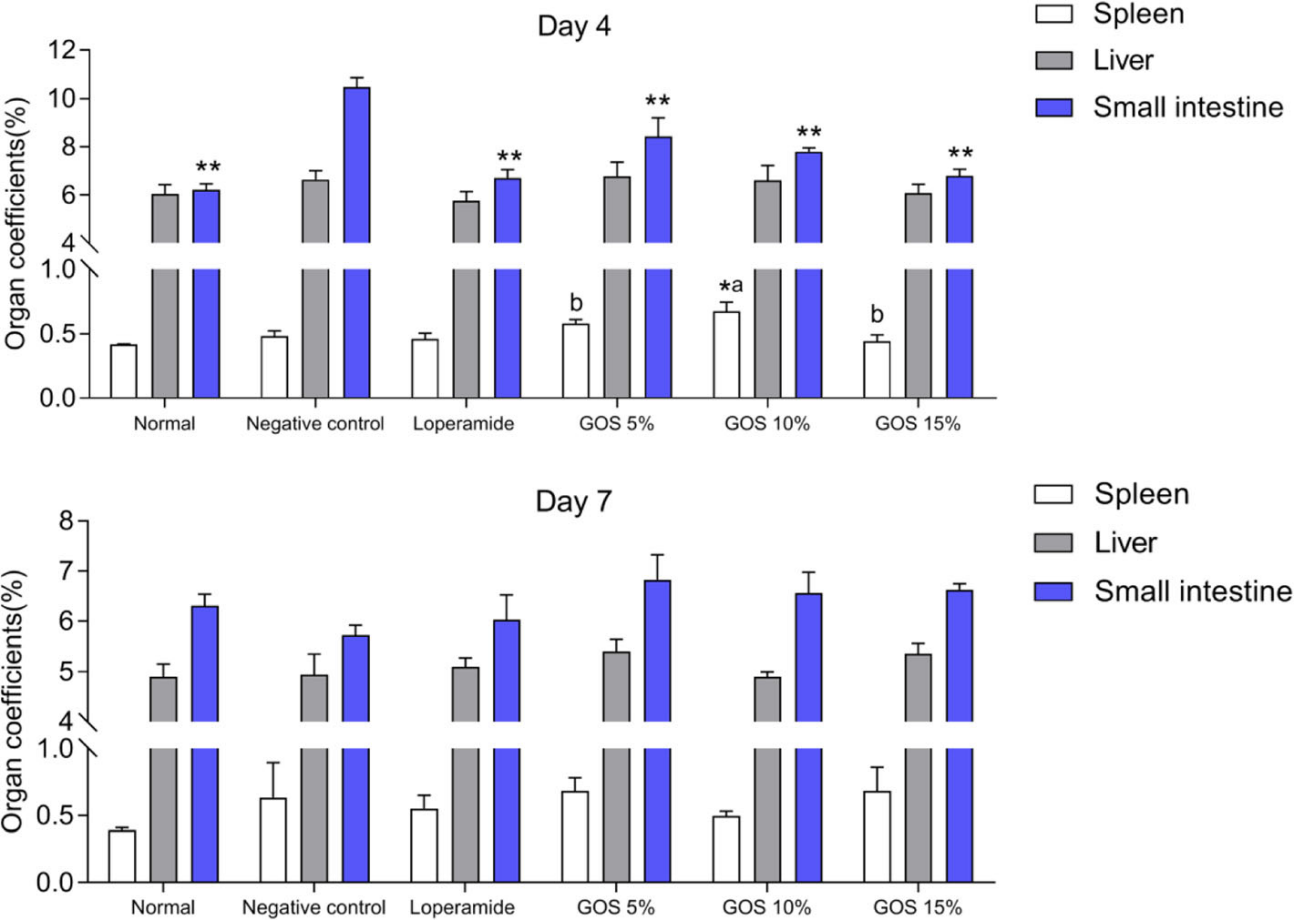
Organ coefficients in each group. Normal, the uninfected-untreated control. Negative control, the infected-untreated control. Loperamide, the infected group with Loperamide-treatment (10 mg/kg). The 5, 10 and 15% GOS were the infected groups treated with corresponding concentration of GOS at a dose of 10 mL/kg, respectively. ^*^ *P* < 0.05 and ^**^ *P* < 0.01, compared with the negative control. ^a, b^ Different letters indicate significant differences existed among the three GOS groups (*P* < 0.05). Data are presented as mean ± S.E.M. *n* = 6

The results of organ coefficients were showed in Fig. 3. On day 4, spleen coefficient of the 10% GOS-treated group was significantly enhanced than that of negative control (*P* < 0.01). ETEC can cause diarrhea, inducing excessive secretion and intestinal motility [30]. Small intestine coefficient reflected the fluid secretion of small intestine [21]. GOS reduced the fluid secretion dose-dependently. Without treatment, infection induced significant increased small intestine coefficient in comparison with the treated groups (*P* < 0.01), indicating that GOS could decrease the fluid secretion caused by ETEC infection in mice. On day 7, mice in each group that survived from ETEC infection showed no significant differences. The organ coefficients of loperamide and GOS were numerically close to the normal group, which may due to the anti-secretory and anti-motility activities [21].

### Levels of cytokines and immunoglobulins

**Fig. 4:**
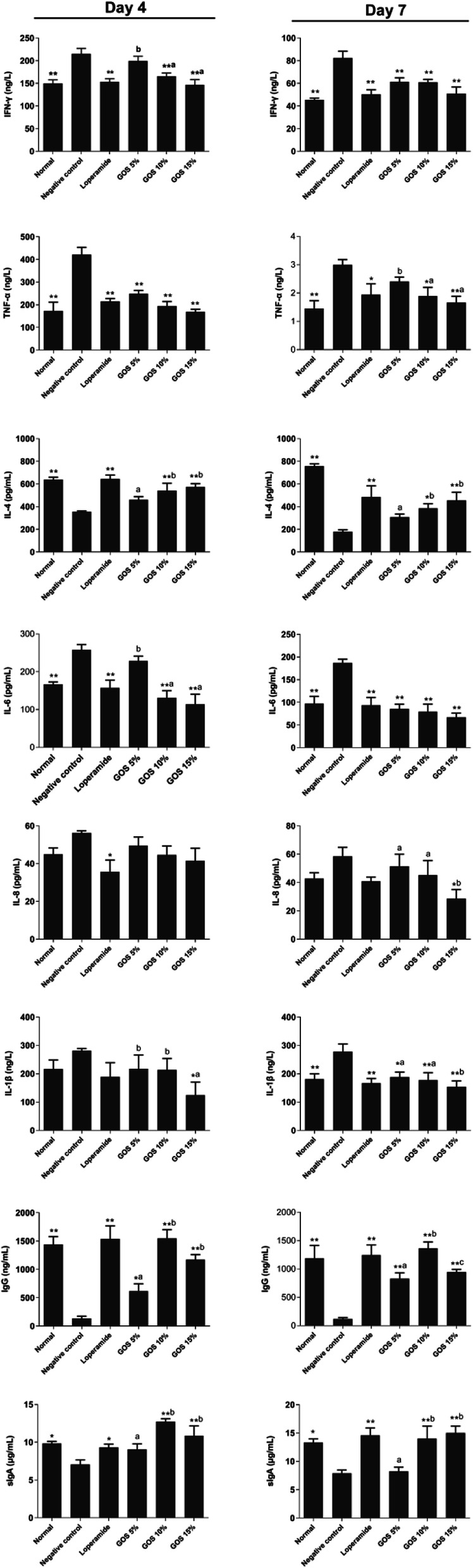
The concentrations of IFN-γ, TNF-α, IL-1β, IL-4, IL-6, IL-8, IgG and sIgA in serum. Normal, the uninfected-untreated control. Negative control, the infected-untreated control. Loperamide, the infected group with Loperamide-treatment (10 mg/kg). The 5, 10 and 15% GOS were the infected groups treated with corresponding concentration of GOS at a dose of 10 mL/kg, respectively. * *P* < 0.05 and ** *P* < 0.01, compared with the negative control. ^a, b, c^ Different letters indicate significant differences existed among the three GOS groups (*P* < 0.05). Data are presented as mean ± S.E.M, *n* = 6

The concentrations of serum cytokines (IFN-γ, TNF-α, IL-1β, IL-4, IL-6 and IL-8) and immunoglobulins (IgG and sIgA) were shown in Fig. 4. After infection, the levels of IL-6, IFN-γ and TNF-α on days 4 and 7 in the untreated group were significantly increased (*P* < 0.01), and treatment with loperamide and GOS could inhibit the secretion of IFN-γ and TNF-α. ETEC infection significantly decreased the levels of IL-4 (*P* < 0.01), IgG (*P* < 0.01) and sIgA (*P* < 0.05) on days 4 and 7 in serum, and treatment with loperamide and GOS could recover the levels. The level of IL-8 on days 4 and 7 was not significantly changed after infection. The level of IL-1β in the untreated group was not significantly changed on day 4, but significantly increased on day 7 in comparison with normal group (*P* < 0.01). After treatment with loperamide and GOS, it declined to the normal level. The GOS could dose-dependently decrease the levels of proinflammatory cytokines (IFN-γ, TNF-α, IL-1β, IL-6 and IL-8) and increase the level of anti-inflammatory cytokine IL-4.

### Determination of selected cecum microbiota

The results of selected cecum microbiota in mice were listed in Table 3. On days 4 and 7, the number of *E.coil* was significantly increased in the infected groups (*P* < 0.01). After treatment with loperamide and GOS, It was significantly decreased in comparison with the untreated group (*P* < 0.05). The number of *Enterococcus*, *Lactobacilli* and *Bifidobacteria* were not significantly changed on day 4. The number of *Enterococcus* was significantly increased without treatment (*P* < 0.05) on day 7; loperamide and GOS could recover the number of *Enterococcus* to normal level. The number of *Lactobacilli* and *Bifidobacteria* were significantly increased in the treated groups on day 7, which were significantly higher than those in the normal group.

**Table 3:** The counts of *E.coil*, *Enterococcus*, *Lactobacilli* and *Bifidobacteria* in cecum

Group	Day 4				Day 7			
	*E.coil*	*Enterococcus*	*Lactobacilli*	*Bifidobacteria*	*E.coil*	*Enterococcus*	*Lactobacilli*	*Bifidobacteria*
Normal	5.45 ± 0.10 ^**^	5.21 ± 0.17	8.19 ± 0.08	8.34 ± 0.13	5.55 ± 0.17 ^**^	5.27 ± 0.29 ^*^	8.76 ± 0.49 ^*^	8.87 ± 0.30 ^*^
Negative control	9.00 ± 0.09	5.96 ± 0.61	8.55 ± 0.46	8.38 ± 0.91	7.24 ± 0.56	6.07 ± 0.47	7.77 ± 0.38	8.01 ± 0.62
Loperamide	7.27 ± 0.67 ^**^	5.83 ± 0.57	8.83 ± 0.25	8.30 ± 0.93	6.20 ± 0.84 ^**^	5.93 ± 0.38	9.61 ± 0.28 ^**^	9.40 ± 0.32 ^**^
GOS 5%	8.40 ± 0.34 ^*a^	6.29 ± 0.61	8.33 ± 0.80	8.40 ± 0.54	5.38 ± 0.13 ^**a^	5.36 ± 0.32 ^*^	9.03 ± 0.50 ^**^	9.35 ± 0.38 ^**^
GOS 10%	7.44 ± 0.32 ^**b^	6.13 ± 0.67	9.02 ± 0.36	9.54 ± 0.31	5.56 ± 0.38 ^**a^	5.31 ± 0.32 ^*^	8.89 ± 1.02 ^*^	9.74 ± 0.30 ^**^
GOS 15%	8.15 ± 0.26 ^**a^	6.26 ± 0.30	8.93 ± 0.59	9.64 ± 0.44	6.43 ± 0.34 ^*b^	5.94 ± 0.60 ^*^	9.31 ± 0.51 ^**^	10.15 ± 0.55 ^**^

Normal, the uninfected-untreated control. Negative control, the infected-untreated control. Loperamide, the infected group with Loperamide-treatment (10 mg/kg). The 5, 10 and 15% GOS were the infected groups treated with corresponding concentration of GOS at a dose of 10 mL/kg, respectively. ^*^ *P* < 0.05 and ^**^ *P* < 0.01, compared with the negative control. ^a, b^ Different letters indicate significant differences existed among the three GOS groups (*P* < 0.05). Data are presented as mean ± S.E.M, *n* = 6

### Histopathological examination of colon

**Fig. 5:**
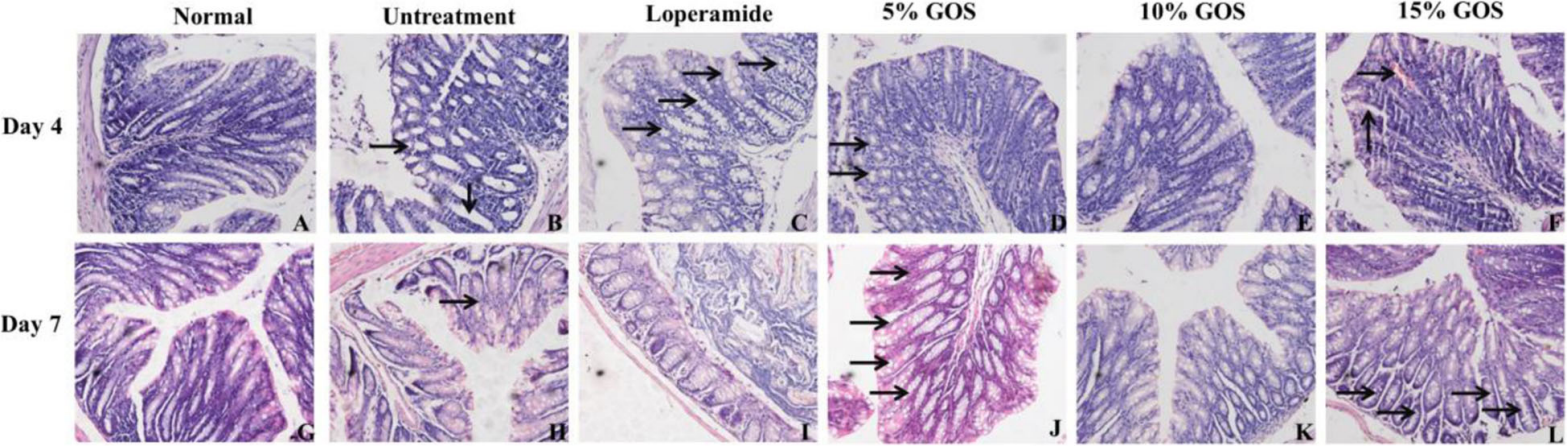
The histological appearance of colon. On day 4 (**a**-**f**), the structure of mucous layer was destroyed and goblet cells disappeared in the untreated group (**b**, denoted by arrowhead); the mice treated with loperamide showed increased number of goblet cells (**c**, denoted by arrowhead); in the 5% GOS group, some of goblet cells were fused (**d**, denoted by arrowhead); in the 10% GOS group, no lesions were observed (**e**); after treated with 15% GOS, congestion and enlarged goblet cells were observed (**f**, denoted by arrowhead). On day 7 (**g**-**l**), in the untreated group, fused goblet cells were observed (**h**, denoted by arrowhead); treatment with loperamide (**i**) and 10% GOS (**k**), the mice showed normal structure of colon; in the 5% GOS and 15% GOS groups, the number of goblet cells was increased (**j** and **l**, denoted by arrowhead). **a** and **g**, normal structure of colon. Normal, the uninfected-untreated control. Untreatment, the infected-untreated control. Loperamide, the infected group with Loperamide-treatment (10 mg/kg). The 5, 10 and 15% GOS were the infected groups treated with corresponding concentration of GOS at a dose of 10 mL/kg, respectively

The histopathological changes of colon were shown in Fig. 5. ETEC infection induced diarrhea on day 4, thus the structure of mucous layer was destroyed and goblet cells disappeared in the untreated group (Fig. 5b). The mice treated with loperamide showed increased number of goblet cells (Fig. 5c). In the 10% GOS group, no lesions were observed (Fig. 5e). In the 5% GOS group, some of goblet cells were fused (Fig. 5d). Treated with 15% GOS, congestion and enlarged goblet cells were observed (Fig. 5f). When mice survived from *E. coli* O101 infection (Day 7, Fig. 5g-l), the structure of mucous layer recovered in different degrees. In the untreated group, fused goblet cells were observed (Fig. 5h). Treatment with loperamide (Fig. 5i) and 10% GOS (Fig. 5k), the mice showed normal structure of colon. In the 5 and 15% GOS groups, the number of goblet cells was increased (Fig. 5j and l).

## Discussion

It is well known that traditional herbal medicines can inhibit bacteria growth and be used in the treatment of infectious diseases. *Galla chinensis* has been traditionally used for a long time for the treatment of diarrhea, prolonged coughing and spontaneous perspiration in China [31]. Previously, we had found that GOS showed significant antidiarrheal activity in a castor oil-induced diarrhea model in mice [21]. In this study, we performed antibacterial experiments in ETEC-infected mice in order to find the potential of GOS for treatment of infectious diarrhea.

Throughout the in vivo study, the changes of body weight were observed; 10% GOS had improved more than other groups. *Galla Rhois* tannins extract had been reported to alter growth performance in post-weaning piglets [32], which supported our results indeed. Spleen is main immune organ, and spleen coefficients in 10% GOS group was significantly enhanced compared with the untreated group. The proliferation of lymphocytes in the body often induces the increased immune organ weight that directly reflects the state of immune response [33], suggesting that GOS might promote the proliferation of lymphocytes. The proportional relationship between organ weight and body is required for the valid use of the organ-to-body weight ratio [34]. In the present study, the small intestine ratios in the infected groups were significantly changed at 4 dpi, and the slope term (*p*-value of 0.026) of the proportional relationship between small intestine weight and body is significantly different from 0 and the intercept (*p*-value of 0.153) is not, which suggested that relationship between the small intestine weight and body weight is proportional and the small intestine ratio is optimal to evaluate the toxicity [34]. These results suggested that the ETEC infection produced toxic to the small intestine. After treated with GOS, it was significantly decreased after treatment with GOS, indicating that GOS could inhibit fluid secretion, which was similar to our previous study [26].

ETEC infection always causes inflammation. Cytokines are the key signals in the immune system and are known to participate in inflammation [35]. The intensity of pro-inflammatory response is associated with tissue damage that is neutralized by the release of anti-inflammatory cytokines [36]. The function of the intestinal barrier may be regulated by a network of multiple cytokines, including ILs, IFNs and TNF-α [37]. The cytokines resulted in immune activation and tissue inflammation are thought to be important in the initiation and/or development of several intestinal and systemic diseases [38]. It is said that IFN-γ promotes immune responses by activating macrophages, and inhibits signaling pathways downstream of anti-inflammatory cytokines to antagonize their suppressive functions [39]. IL-1β is markedly elevated in intestinal mucosa under inflammatory conditions [38]. Excessive secretion of IL-6 and dysregulation of the signaling pathway may play a major role in the pathogenesis of many diseases [38]. TNF-α exerts its pro-inflammatory effects through increased productions of IL-1β and IL-6, expressions of adhesion molecules, proliferation of fibroblasts and procoagulant factors, as well as initiation of cytotoxic, apoptotic and acute-phase responses and inhibition of apoptosis [35]. IL-8 plays a causative role in acute inflammation by recruiting and activating neutrophils [40]. IL-4 is a stimulatory molecule for B and T cells and has known immunosuppressive effects in the intestine, which may decrease IFN-γ [35]. In these studies, *E. coli* infection increased the concentrations of pro-inflammatory cytokines (IFN-γ, TNF-α, IL-1β, IL-6 and IL-8) and decreased the concentrations of anti-inflammatory cytokine (IL-4), indicating that *E. coli* could induce inflammation in mice through regulation of cytokines. However, levels of pro-inflammatory cytokines had been reduced by GOS, while the levels of the anti-inflammatory cytokine had been increased. Tannin-rich plant had been reported to down-regulate the levels of cytokines [41]. As a tannin-rich product, the activity of GOS was closely associated with the inhibition of inflammation caused by infection of *E. coli* O101.

GOS could enrich the concentrations of IgG in serum and sIgA in the terminal ileum. Resistance to infection is due, in part, to the presence of sufficient levels of serum and secretory immunoglobulins, especially the antigen-specific antibodies IgG and sIgA [42]. IgG has specific antibody activity against microbial agents or antigens [42]. sIgA is produced at mucosal surfaces contributes to host defense against intestinal pathogens [43]. Therefore, the increase of IgG and sIgA induced by GOS is meaningful to body infection.

Intestinal bacteria regulate the immune system, the development of the gut-associated immune system and epithelial cell functions. One of the most important functions of the intestinal flora is the prevention of bacterial overgrowth and susceptibility to infection with extraneous pathogenic organisms [44]. The current study showed that ETEC challenge enhanced the number of *E.coil* in cecum flora. In those conditions, GOS significantly reduced the number of *E.coil*. GOS also significantly enhanced the number of probiotics like *Lactobacilli* and *Bifidobacteria*. *Bifidobacterium* and *Lactobacillus* are the most widely used probiotic bacteria, exerting health-promoting properties [45]. It is believed that probiotics appear to have clear beneficial effects in shortening the duration and reducing stool frequency in acute infectious diarrhea [46]. The increase of probiotics may be associated with antidiarrheal activity of GOS.

Toxigenic bacteria, like ETEC, which produce enterotoxins able to cross the intestinal mucosa. Therefore, a feature of ETEC infection is effacing of the intestinal mucosa [37]. Our results suggested that *E. coli* O101 infection destroyed the structure of mucous layer and decreased goblet cells in colon, while GOS protected or recovered the intestinal mucosa and the number of goblet cells. Colonic mucus systems is anchored to the goblet cells, which functions as separation of the luminal content, especially bacteria, from direct contact with the epithelial cells [47]. In this study, GOS prevented the damage of structure of the colonic mucosa by ETEC, providing an evidence of its antibacterial activity.

Survival rate revealed that oral administration of GOS increased the protective efficacy against *E. coli* O101, which may be due to the GOS altering the levels of cytokines and immunoglobulin, regulating the intestinal bacteria and protecting the structure of colonic mucosa. The GOS also reduced the secretion fluid in the small intestine, which might relieve the symptoms of ETEC infection.

Accumulated evidences have demonstrated that tannins showed inhibitory effects on enterotoxin-induced secretory diarrheas, which are involved in the hyperactivation of CFTR channel function. Tannins have been shown to inhibit CFTR-dependent Cl^−^ secretion in Caco-2, FRT, T84 and HT29-CL19A cells [48]. It has also been shown that tannins could inhibit enterotoxin production and activities [49]. In the previous study, GOS mainly consisted of tannins can inhibit castor oil-induced diarrhea in mice through reducing the fluid secretion. In the present study, GOS was also proven to inhibit ETEC-induced diarrhea in mice. Thus, whether the antidiarrheal activity of GOS against two types of diarrhea mode was related to inhibition of CFTR function needs to be further clarified.

## Conclusion

GOS has therapeutic and protective effects against ETEC infection by altering the levels of cytokines and immunoglobulins, regulating the intestinal bacteria and protecting the structure of colonic mucosa. GOS is a potent antidiarrheal preparation, which is expected to be applied to treat ETEC infection. Further study should be conducted to elucidate the antidiarrheal mechanism of GOS.

## Data Availability

The datasets used and/or analyzed during the current study are available from the corresponding author upon reasonable request.

## Abbreviations

GOS: *Galla Chinensis* oral solution
ETEC: Enterotoxigenic *Escherichia coli*
LD_50_: Median lethal dose
CFTR: Cystic fibrosis transmembrane conductance regulator
